# Dysfunctional peroxisomal lipid metabolisms and their ocular manifestations

**DOI:** 10.3389/fcell.2022.982564

**Published:** 2022-09-07

**Authors:** Chuck T. Chen, Zhuo Shao, Zhongjie Fu

**Affiliations:** ^1^ Department of Nutritional Sciences, Temerty Faculty of Medicine, University of Toronto, Toronto, ON, Canada; ^2^ Post-Graduate Medical Education, University of Toronto, Toronto, ON, Canada; ^3^ Division of Clinical and Metabolic Genetics, the Hospital for Sick Children, University of Toronto, Toronto, ON, Canada; ^4^ The Genetics Program, North York General Hospital, University of Toronto, Toronto, ON, Canada; ^5^ Department of Ophthalmology, Boston Children’s Hospital, Harvard Medical School, Boston, MA, United States

**Keywords:** peroxisome, retinal lipids, fatty acid oxidation, retinal dystrophy, retinopathy

## Abstract

Retina is rich in lipids and dyslipidemia causes retinal dysfunction and eye diseases. In retina, lipids are not only important membrane component in cells and organelles but also fuel substrates for energy production. However, our current knowledge of lipid processing in the retina are very limited. Peroxisomes play a critical role in lipid homeostasis and genetic disorders with peroxisomal dysfunction have different types of ocular complications. In this review, we focus on the role of peroxisomes in lipid metabolism, including degradation and detoxification of very-long-chain fatty acids, branched-chain fatty acids, dicarboxylic acids, reactive oxygen/nitrogen species, glyoxylate, and amino acids, as well as biosynthesis of docosahexaenoic acid, plasmalogen and bile acids. We also discuss the potential contributions of peroxisomal pathways to eye health and summarize the reported cases of ocular symptoms in patients with peroxisomal disorders, corresponding to each disrupted peroxisomal pathway. We also review the cross-talk between peroxisomes and other organelles such as lysosomes, endoplasmic reticulum and mitochondria.

## 1 Introduction

**FIGURE 1:**
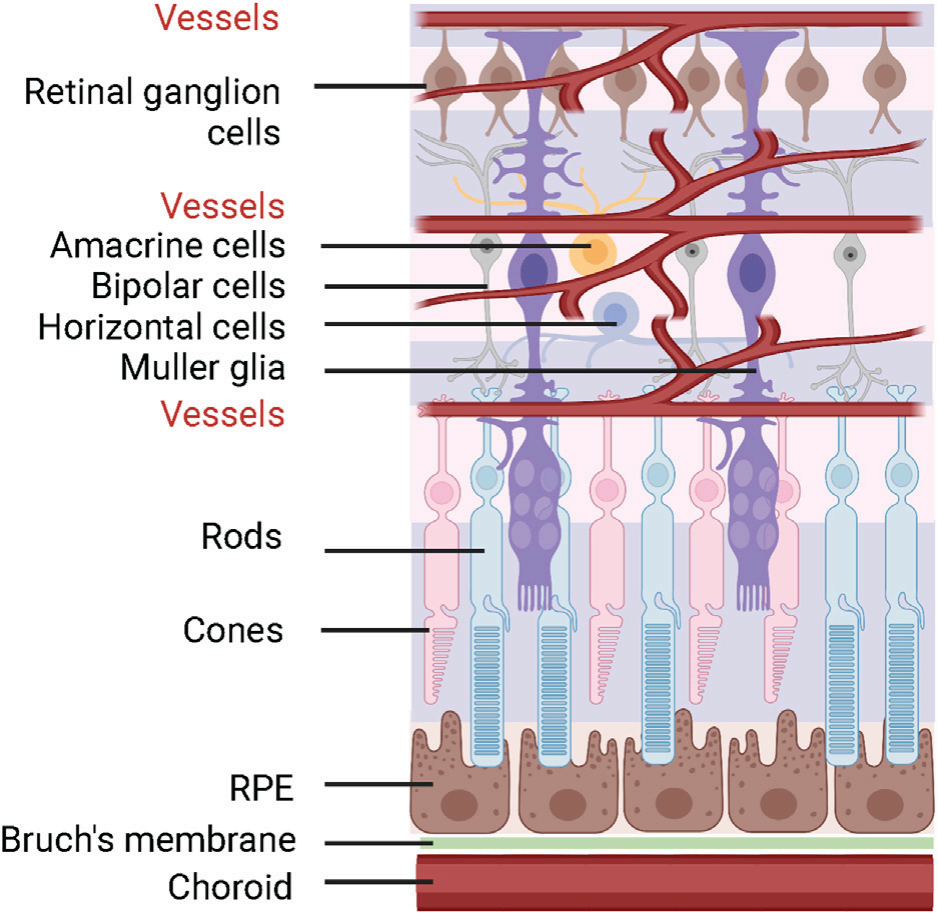
Schematics of retinal structure. The neural retina is supplied by retinal and choroidal vasculature. Photoreceptors (rods and cones) are supported by RPE and Müller glia cells. The figure was generated using Biorender (license # LX24BJEUXV).

Peroxisomes are membrane bound organelles found in all eukaryotic cells and heavily involved in α and β-oxidation of lipids, glyoxylate detoxification, amino acid degradation, as well as reactive oxygen species (ROS) and reactive nitrogen species (RNS) metabolism. Peroxisomes are also essential in plasmalogen, docosahexaenoic acid (DHA) and bile acid synthesis. There are abundance of enzymes and transporter proteins associated with peroxisomes that are responsible for peroxisome biogenesis, transportation across peroxisomal membrane, and metabolism. Loss of function in various proteins due to pathogenic genetic variants lead to multisystem disorders in human. As these mutations cause the potential for severe and early onset neurological, ophthalmological, craniofacial, musculoskeletal, and hepatorenal damages, they pose significant health risks and burden on patients and their families (Steinberg et al., 1993). Recent studies have also suggested that peroxisomes are involved in common neurodegenerative diseases such as Alzheimer’s disease, Parkinson’s disease, and multiple sclerosis (Lizard et al., 2012; Wojtowicz et al., 2020). Since neurodegeneration is associated with oxidative stress, lipid peroxidation, and cholesterol auto-oxidation, modifications of peroxisomal functions have been proposed as a potential mechanism for the altered metabolome. Retina is rich in lipids and highly metabolically demanding. Photoreceptors are very high in mitochondria, and nursed by retinal pigment epithelium (RPE) which uptakes nutrients from choroidal blood vessels and Müller glia which extends across the retinal layers and safeguards the blood-retinal barrier (Figure 1). This unique anatomical landscape and high metabolic demand made retina a vulnerable tissue prone to damage from metabolic insults such as peroxisomal disorders. In this review, we focus on the peroxisomal metabolism pathways that are involved in retinal health and the ocular dysfunction associated with peroxisomal disorders.

## 2 Peroxisome metabolic functions

**FIGURE 2:**
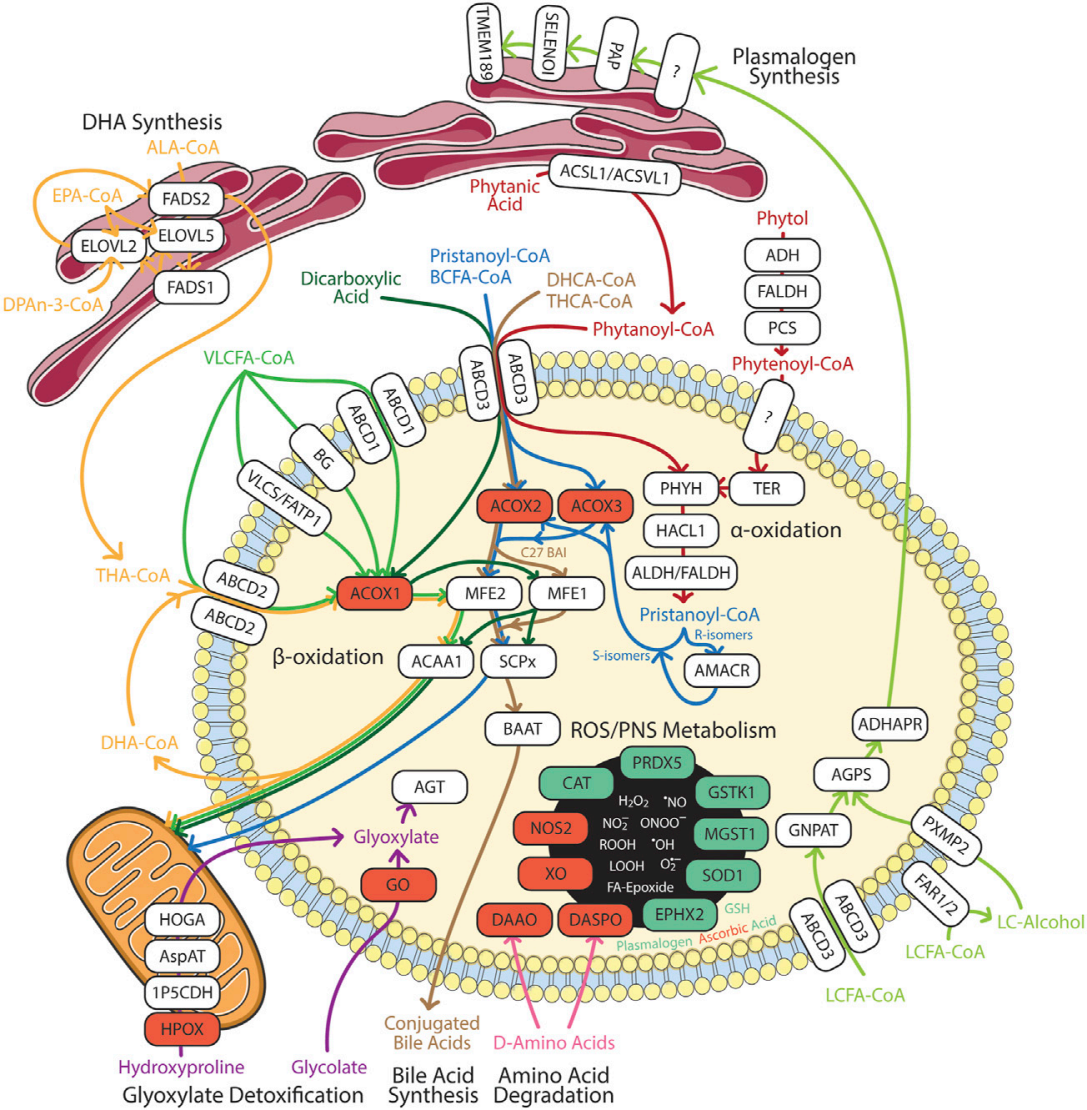
Summary of peroxisomal functions in humans. Enzymes involved in the catabolic and anabolic pathways in white boxes. Red box represents ROS/RNS producing enzymes. Green box represents anti-oxidation enzymes in redox homeostasis. 1P5CDH, Δ^1^-pyrroline-5-carboxylate dehydrogenase; ABCD, ATP-binding cassette sub-family D; ACAA, 3-oxoacyl thiolase; ACOX, acyl-CoA oxidase; ACSL, long chain acyl-CoA synthetase; ACSVL, very long chain acyl-CoA synthetase; ADH, alcohol dehydrogenase; ADHAPR, acyl/alkyl DHAP reductase; AGPS, alkyl-glycerone phosphate synthase; AGT, alanine:glyoxylate aminotransferase; ALA, α-linolenic acid; ALDH, aldehyde dehydrogenase; AMACR, α-methylacyl-CoA racemase; AspAT, aspartate aminotransferase; BAAT, bile acid-CoA:amino acid N-acyltransferase; BCFA, branched chain fatty acids; BG, bubblegum; CAT, catalase; DAAO, D-amino acid oxidase; DASPO, D-aspartate oxidase; DHA, docosahexaenoic acid; DHCA, 3α,7α-dihydroxy-5β-cholestanoic acid; DPAn-3, n-3 docosapentaenoic acid; ELOVL, fatty acid elongase; EPA, eicosapentaenoic acid; EPHX2, epoxide hydrolase 2; FADS, fatty acid desaturase; FALDH, fatty aldehyde dehydrogenase; FAR, fatty acyl-CoA reductase; FATP, fatty acid transport protein; GNPAT, glycerone-phosphate O-acyltransferase; GO, glycolate oxidase; GSH, glutathione; GSTK, glutathione transferase kappa; HACL, 2-hydroxyacyl-CoA lyase; HOGA, 4-hydroxy-2-oxoglutarate aldolase; HPOX, hydroxyproline oxidase; LCFA, long chain fatty acids; MFE, multifuctional enzyme; MGST, microsomal glutathione S-transferase; NOS2, inducible nitric oxide synthase; PAP, phosphatidic acid phosphatase; PCS, phytenoyl-CoA synthetase; PHYH, phytanoyl-CoA 2-hydroxylase; PRDX5, peroxiredoxin 5; PXMP2, peroxisomal membrane protein 2; SELENOI, selenoprotein 1 (ethanolamine phosphotransferase 1); SCPx, SCP-2/3-ketoacyl-CoA thiolase; SOD1, superoxide dismutase; TER, trans-2-enoyl-CoA reductase; THA, tetracosahexaenoic acid; THCA, 3α,7α, 12 α-trihydroxy-5β-cholestanoic acid; TMEM189, plasmanylethanolamine desaturase; VLCFA, very long chain fatty acids; VLCS, very long chain acyl-CoA synthetase; XO, xanthine oxidase. The Figure was partly generated using Servier Medical Art, provided by Servier, licensed under a Creative Commons Attribution 3.0 unported license.

Peroxisomes are essential for biosynthesis of DHA, plasmalogen and bile acids, as well as degradation and detoxification of very-long-chain fatty acids (VLCFAs), branched-chain fatty acids (BCFAs), dicarboxylic acids, reactive oxygen/nitrogen species (ROS/RNS), glyoxylate, and amino acids (Islinger et al., 2010; Islinger et al., 2018). In this section, we summarize the current updates on these pathways (Figure 2) and the eye diseases associated with gene mutation in these pathways (Table 1). The detailed eye symptoms are discussed in Section 3. The abbreviations are listed in Table 2.

**TABLE 1:** Summary of common gene mutations and ocular symptoms in peroxisomal disorders.

Gene	Function	Peroxisomal disorders	Ocular symptoms
ACOX1	Peroxisomal β-oxidation	Single enzyme deficiency	Nystagmus, optic atrophy, tapetoretinal degeneration, pigmentary retinopathy, flattened or absent electroretinogram, and absence of flash-evoked visual responses
AGPS	Plasmalogen synthesis	RCDP	None reported
AGXT	Detoxification of glyoxylate	Single enzyme deficiency	Optic atrophy, choroidal neovascularization, and crystalline retinopathy
ALDH3A2/FALDH	Phytenic acid production	Single enzyme deficiency	Photophobia, superficial corneal opacity, glistening white dots in fundus, central retinal thinning/macular degeneration, macular window defects without leakage, heterogeneous macular autofluorescence with crystals, and retinal pigment epithelial atrophy
AMACR	Bile acid synthesis/α-oxidation	Single enzyme deficiency	Variable pigmentary retinopathy
FAR1	Plasmalogen synthesis	RCDP	Cataracts
GNPAT	Plasmalogen synthesis	RCDP	Cataract
HACL1	Peroxisomal α-oxidation	No associated syndrome	Not reported
HSD17B4/MFE2	Peroxisomal β-oxidation multifunctional protein-2 (MFP2)	Single enzyme deficiency	Nystagmus, strabismus, abolished electroretinogram, and limited extraocular movements
PEX family genes	Peroxisomal biogenesis	PBD-ZSD	Nystagmus, glaucoma, corneal clouding, cataracts, pigmentary retinopathy, optic disc hypoplasia, abnormal electroretinogram, and macular dystrophy (rare)
PHYH	Peroxisomal α-oxidation	Single enzyme deficiency that mimics ZSD	Nystagmus, cataracts, retinitis pigmentosa, retinal degeneration, night blindness, constriction of visual fields, miosis, and poor pupillary reaction
SCPx/SCP2	branched-chain fatty acids β-oxidation	Single enzyme deficiency	Pathologic saccadic eye movements

**TABLE 2:** Abbreviations.

Abbreviation	Full name
ABCD1/2	ATP binding cassette sub family D members 1/2
ACBD5	acyl-CoA binding domain containing protein 5
ACOX	acyl-CoA oxidase
AGPS	alkyl-glycerone phosphate synthase
AMD	age-related macular degeneration
ARA	arachidonic acid
BCFA	branched-chain fatty acids
CA	cholic acid
CAT	Catalase
CDCA	chenodeoxycholic acid
CNS	central nervous system
CPTI	carnitine palmitoyltransferase I
CYP	cytochrome P450
DHA	docosahexaenoic acid
DHAP	dihydroxyacetone-phosphate
DHCA	3α,7α-dihydroxy-5β-cholestanoic acid
DPA	docosapentaenoic acids
EPA	eicosapentaenoic acid
ER	endoplasmic reticulum
ERG	electroretinogram
FALDH	fatty aldehyde dehydrogenase
FAR	fatty acyl-CoA reductase
GNPAT	glycerone-phosphate O-acyltransferase
HACL1	2-hydroxyacyl-CoA lyase
HOGA	4-hydroxy-2-oxoglutarate aldolase
MFE	multifunctional enzymes
PBD	peroxisomal biogenesis disorders
PHYH	phytanoyl-CoA 2-hydroxylase
Pls	plasmalogen
PTS1	peroxisomal targeting signal type 1
RCDP	rhizomelic chondrodysplasia punctata
ROP	retinopathy of prematurity
ROS/RNS	reactive oxygen/nitrogen species
RPE	retinal pigment epithelium
SCPx	SCP-2/3-ketoacyl-CoA thiolase
THCA	3α,7α, 12 α-trihydroxy-5β-cholestanoic acid
VLCFA	very-long-chain fatty acids
X-ALD	X-linked adrenoleukodystrophy
ZSD	Zellweger Spectrum Disorder

### 2.1 Catabolic function: β-oxidation of monocarboxylic fatty acids and dicarboxylic acids

**FIGURE 3:**
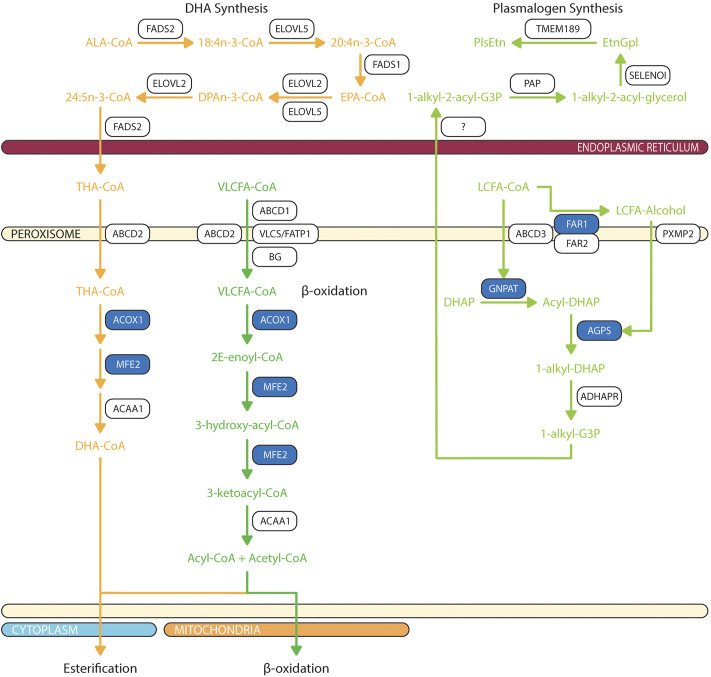
Biochemical pathways of peroxisomal lipid synthesis and degradation. Blue box represents enzymes with published pathogenic variants resulting in ocular manifestations in humans. ABCD, ATP-binding cassette sub-family D; ACAA, 3-oxoacyl thiolase; ACOX, acyl-CoA oxidase; ADHAPR, acyl/alkyl DHAP reductase; AGPS, alkyl-glycerone phosphate synthase; ALA, α-linolenic acid; BG, bubblegum; DHA, docosahexaenoic acid; DHAP, dihydroxyacetone phosphate; DPAn-3, n-3 docosapentaenoic acid; ELOVL, fatty acid elongase; EPA, eicosapentaenoic acid; EtnGpl, ethanolamine glycerophospholipids; FADS, fatty acid desaturase; FAR, fatty acyl-CoA reductase; FATP, fatty acid transport protein; G3P, glycerol-3-phosphate; GNPAT, glycerone-phosphate O-acyltransferase; LCFA, long chain fatty acids; MFE, multifuctional enzyme; PAP, phosphatidic acid phosphatase; PlsEtn, ethanolamine plasmalogens; PXMP2, peroxisomal membrane protein 2; SELENOI, selenoprotein 1 (ethanolamine phosphotransferase 1); THA, tetracosahexaenoic acid; TMEM189, plasmanylethanolamine desaturase; VLCFA, very long chain fatty acids; VLCS, very long chain acyl-CoA synthetase. The figure was partly generated using Servier Medical Art.

**FIGURE 4:**
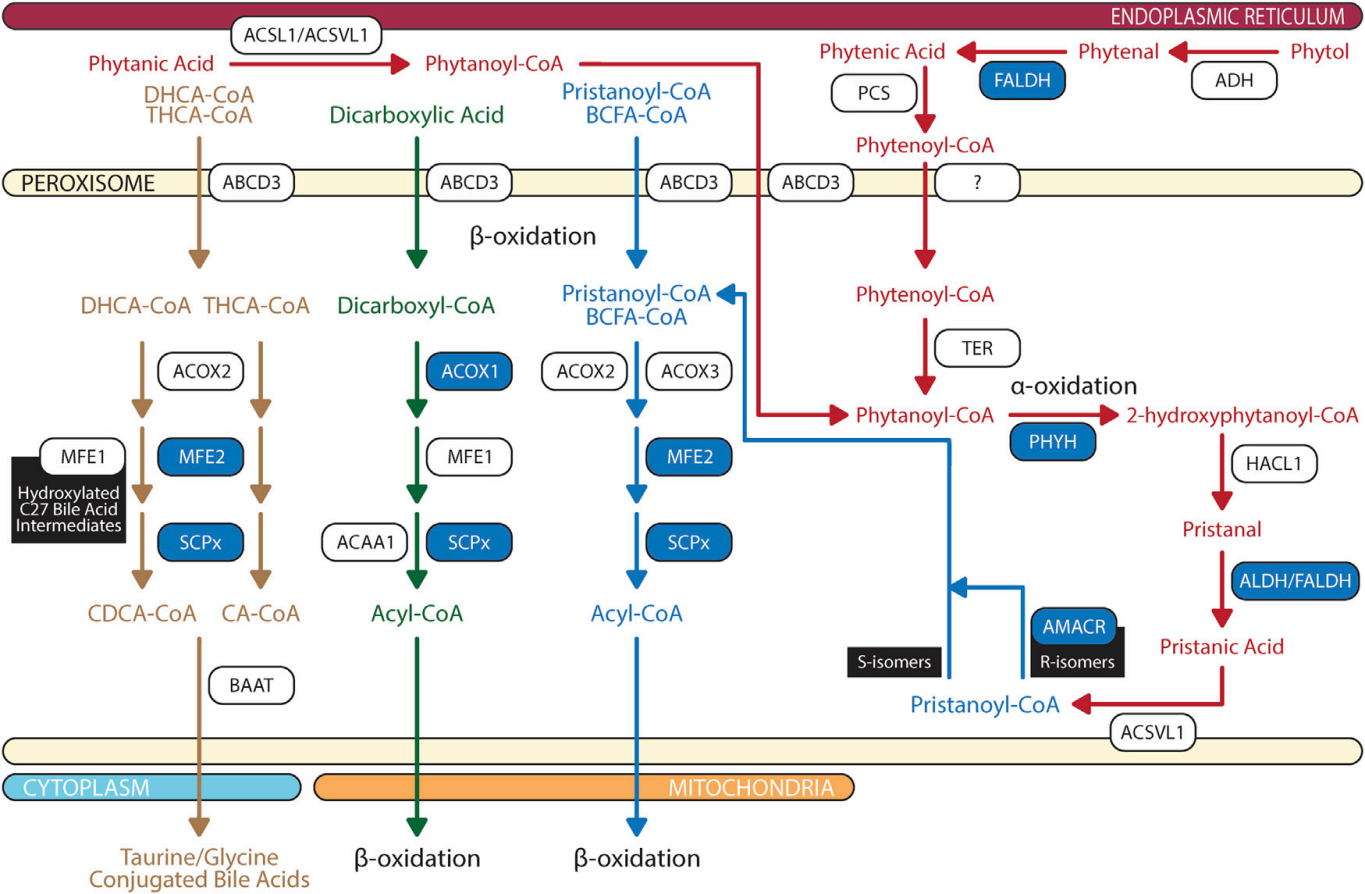
Biochemical pathways of peroxisomal branched chain fatty acid, dicarboxylic acid, and bile acid degradations. Blue box represents enzymes with published pathogenic variants resulting in ocular manifestations in humans. ABCD, ATP-binding cassette sub-family D; ACAA, 3-oxoacyl thiolase; ACOX, acyl-CoA oxidase; ADH, alcohol dehydrogenase; ALDH, aldehyde dehydrogenase; AMACR, α-methylacyl-CoA racemase; AspAT, aspartate aminotransferase; BAAT, bile acid-CoA:amino acid N-acyltransferase; BCFA, branched chain fatty acids; CA, cholic acid; CDCA, chenodeoxycholic acid; DHCA, 3α,7α-dihydroxy-5β-cholestanoic acid; FALDH, fatty aldehyde dehydrogenase; HACL, 2-hydroxyacyl-CoA lyase; MFE, multifuctional enzyme; PCS, phytenoyl-CoA synthetase; PHYH, phytanoyl-CoA 2-hydroxylase; SCPx, SCP-2/3-ketoacyl-CoA thiolase; TER, trans-2-enoyl-CoA reductase; THCA, 3α,7α, 12 α-trihydroxy-5β-cholestanoic acid. The figure was partly generated using Servier Medical Art.

Peroxisomal β-oxidation of monocarboxylic fatty acids and dicarboxylic acids are ubiquitous within the animal kingdom (Ferdinandusse et al., 2004; Wanders, 2014). VLCFAs are a family of monocarboxylic fatty acids with greater than 22 carbons. These include saturated, monounsaturated, and polyunsaturated species of fatty acids such as behenic acid (22:0), lignoceric acid (24:0), cerotic acid (26:0), erucic acid (22:1n-9), nervonic acid (24:1n-9), adrenic acid (22:4n-6), docosapentaenoic acids (DPA; 22:5n-6 or 22:5n-3), DHA (22:6n-3), and nisinic acid/tetracosahexaenoic acid (THA; 24:6n-3). Since mitochondria lack expression of very long chain acyl-CoA synthetase (very long chain acyl-CoA synthetase/fatty acid transport protein, VLCS/FATP; bubblegum), and mitochondrial carnitine palmitoyltransferase I (CPTI) preferentially transports long-chain fatty acids (LCFA - 14–20 carbons), VLCFA are largely metabolized by the peroxisome (Gavino and Gavino, 1991; Lageweg et al., 1991; van den Bosch et al., 1992; Reddy and Mannaerts, 1994; Uchiyama et al., 1996; Coe et al., 1999; Smith et al., 1999; Steinberg et al., 1999b; Steinberg et al., 2000; Heinzer et al., 2003; Pridie et al., 2020) (Figure 3). Furthermore, peroxisomal ATP binding cassette sub family D members 1/2 (ABCD1/2) are essential for transport of VLCFA; though the exact mechanism is still debated (van Roermund et al., 2011; Kawaguchi et al., 2021). Long chain dicarboxylic acid are metabolites of monocarboxylic acid ω-oxidation by cytochrome P450 that are products of compromised β-oxidation (Grego and Mingrone, 1995). Dicarboxylic acids are transported to peroxisome via ABCD3 (van Roermund et al., 2014) (Figure 4).

Peroxisomal β-oxidation involves four sequential reactions: 1) dehydrogenation by ACOX, 2 + 3) hydration and dehydrogenation by multifunctional enzymes (MFE), and 4) thiolytic cleavage by 3-ketoacyl-CoA thiolases. The first step of peroxisomal β-oxidation involves ACOX catalyzed acyl-CoA and oxygen to 2E-enoyl-CoA and hydrogen peroxide. In rodents, there are three ACOX with different substrate specificity. Generally, ACOX1 (straight chain-CoA oxidase) has high affinity for CoA esters of medium chain fatty acids, LCFA, and VLCFA, whereas ACOX2 (pristanoyl-CoA oxidase) and ACOX3 (cholestanoyl-CoA oxidase) prefer CoA esters of BCFA, 3α,7α-dihydroxy-5β-cholestanoic acid (DHCA), and 3α,7α, 12 α-trihydroxy-5β-cholestanoic acid (THCA), respectively (Bieber et al., 1981; Vamecq, 1987; Vanhove et al., 1993). In contrast, human ACOX2 dehydrogenate BCFA, DHCA and THCA while human ACOX3 metabolizes BCFA (Ferdinandusse et al., 2018).

Following ACOX, MFE catalyze the hydration and dehydrogenation of 2E-enoyl-CoA to chiral 3-hydroxy-acyl-CoA and 3-ketoacyl-CoA. In mammals, there are two MFE: 1) MFE-1 (L-bifunctional protein; LBP) and 2) MFE-2 (D-bifunctional protein; DBP). While MFE-1 has a broad substrate specificity which can bind CoA esters of fatty acids varying from short to long, hydroxylated C27 DHCA/THCA intermediates, and dicarboxylic acids, it predominantly catalyzes breakdown of medium and long chain dicarboxylic acids (Ferdinandusse et al., 2004; Houten et al., 2012). MFE-2 (coded by the *HSD17B4* gene in human) is essential for the breakdown of VLCFA, BCFA, DHCA, and THCA (Qi et al., 1999; Su et al., 2001; Huyghe et al., 2006).

In the final step of peroxisomal β-oxidation, 3-ketoacyl-CoA thiolases catalyze the cleavage of 3-ketoacyl-CoA to acetyl-CoA and shortened acyl-CoA. In rodents, there are three 3-ketoacyl-CoA thiolases: 1) thiolase A, 2) thiolase B, and 3) SCP-2/3-ketoacyl-CoA thiolase (SCPx). Thiolase A and B have similar profile in substrate specificity, where the preference is given to saturated fatty acid in various chain length. In addition to binding of saturated 3-ketoacyl-CoA esters, SCPx also binds BCFA CoA esters (Poirier et al., 2006). In contrast to rodents, human has two 3-ketoacyl-CoA thiolases: 1) 3-oxoacyl thiolase (ACAA1) and 2) sterol carrier protein X (SCPx—ortholog of rodent SCPx). ACAA1 ligand specificity is restricted to 3-ketoacyl-CoA esters of VLCFA, whereas SCPx prefers 3-oxoacyl-CoA esters of pristanic acid, and DHCA/THCA (Seedorf et al., 1994). ACAA1 is expressed equally or more in neural retina than RPE, while reversely, SCPx expression is more abundant in RPE as compared to neural retina (Das et al., 2019).

### 2.2 Catabolic function: α-oxidation of branched chain fatty acids

BCFAs are a specialized group of saturated fatty acids with mono- or poly-methyl branches on the carbon chain. Monomethyl BCFA are subdivided into iso and anteiso structure. Iso-BCFA have methyl branch on the penultimate carbon which is one carbon from the methyl end; meanwhile anteiso-BCFA have methyl branch on the antepenultimate carbon that is two carbons from the methyl end. Enriched sources of BCFA include ruminant-derived and fermented foods (Taormina et al., 2020). Phytanic acid and its metabolite pristanic acid are common 3-methyl BCFA synthesized in ruminant or derived from phytol that are bioaccumulated in the marine system from phytoplankton chlorophyll (Wanders et al., 2011a; Taormina et al., 2020). In humans, tissue phytanic acid levels are strictly derived from dietary sources as endogenous synthesis are limited (Steinberg et al., 1965).

Dietary phytol is oxidized to phytenal by alcohol dehydrogenase, then converted into phytenic acid by fatty aldehyde dehydrogenase (FALDH) (Rizzo et al., 1999) (Figure 4). Phytenic acid is activated by esterification of CoA to phytenoyl-CoA by phytenoyl-CoA synthetase (PCS) after which it can bind peroxisomal trans-2-enoyl-CoA reductase (TER) and reduced to produce phytanoyl-CoA for subsequent peroxisomal phytanoyl-CoA α-oxidation and pristanoyl-CoA β-oxidation (van den Brink et al., 2005; Gloerich et al., 2006). Reduction of phytenoyl-CoA to phytanoyl-CoA is localized to peroxisome for two reasons: 1) TER is targeted to peroxisome due to attached peroxisomal targeting signal type 1 (PTS1) peptide on the enzyme and 2) mitochondrial CPTI does not catalyze carnitine esterification to phytenoyl-CoA (Wanders et al., 2011a). Though the degradation pathways are characterized, only two enzymes have been identified, FALDH and TER. Further research is required to identify enzymes catalyzing the initial conversion to phytenal and CoA esterification to phytenoyl-CoA.

Like VLCFA, phytanic acid and pristanic acid are ligands for PPARα resulting in upregulation of peroxisomal β-oxidation enzymes and cytochrome P450 4A (CYP4A) enzymes facilitated ω-oxidation of phytanic acid in endoplasmic reticulum (ER) and microsome (Zomer et al., 2000; Wanders et al., 2011b; Kersten, 2014). Under normal conditions, phytanic acid is preferentially catabolized by α-oxidation (Wanders et al., 2011a). Degradation of dietary phytanic acid begins with activation of CoA esterification by phytanoyl-CoA synthetase, ACSL1 and ACSVL1 (Watkins et al., 1994). Phytanoyl-CoA is transported into peroxisome by ABCD3 (Morita and Imanaka, 2012). Hydroxylation of phytanoyl-CoA to 2-hydroxyphytanoyl-CoA is catalyzed by peroxisomal phytanoyl-CoA 2-hydroxylase (PHYH). Similar to TER, PHYH protein sequence includes PTS2; hence after is shuttled to peroxisome (Jansen et al., 1999a). 2-hydroxyphytanoyl-CoA is then cleaved by peroxisomal 2-hydroxyacyl-CoA lyase (HACL1) to pristanal and formyl-CoA (Jansen et al., 1999b; Foulon et al., 1999). Lastly, pristanal is oxidized to pristanic acid by unknown peroxisomal aldehyde dehydrogenase (potential candidate includes FALDH). In contrast to PHYH and HACL1, which are ubiquitously expressed in RPE and neural retina, ABCD3 is more abundantly found in RPE (Das et al., 2019). This suggests that phytanoyl-CoA and pristanoyl-CoA may have alternative route of entry in neural retina as compared to RPE.

Pristanic acid is activated to pristanoyl-CoA by ACSVL1 (Steinberg et al., 1999a). α-oxidation of phytanic acid may produce pristanoyl-CoA enantiomers; therefore, R-isomers are converted to S-isomer by α-methylacyl-CoA racemase (AMACR) in order to continue peroxisomal β-oxidation cycle due to stereospecificity of ACOX2 and ACOX3 (Van Veldhoven et al., 1996). Pristanoyl-CoA is catabolized by ACOX2/3, MFE-2, and SCPx in three rounds of peroxisomal β-oxidation (Verhoeven et al., 1998; Atshaves et al., 2007). Furthermore, recent report found that PMP34 are important in the peroxisomal oxidation of pristanic acid and the export of its metabolites (Van Veldhoven et al., 2020). However, the exact mechanistic details of PMP34’s contribution to pristanic acid degradation requires further examination. AMACR deficiency results in increased plasma pristanic acid levels (Ferdinandusse et al., 2000; Thompson et al., 2009).

### 2.3 Catabolic function: Glyoxylate detoxification and amino acid degradation

**FIGURE 5:**
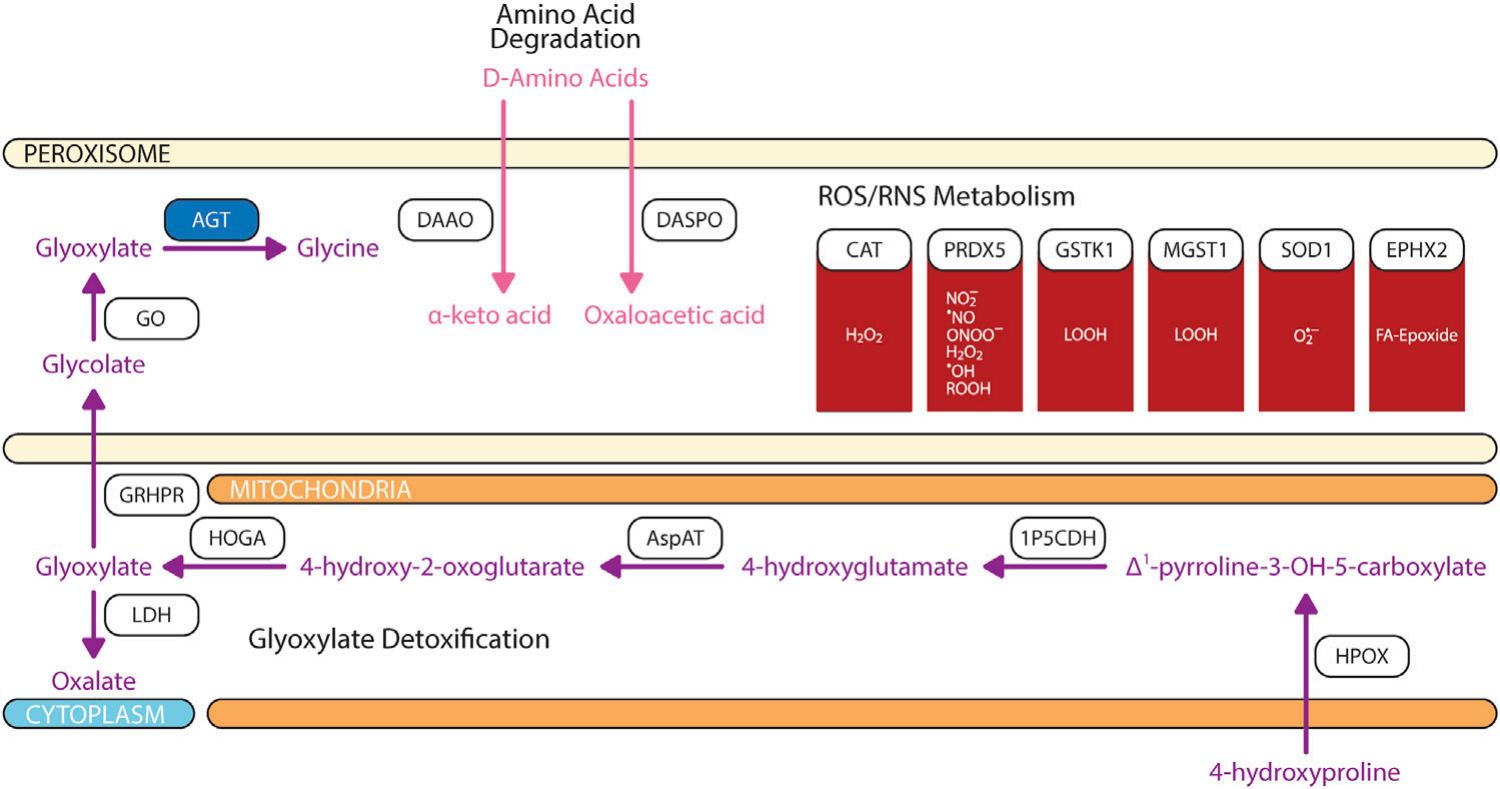
Biochemical pathways of glyoxylate, amino acids, and reactive oxygen/nitrogen species degradations. Blue box represents enzymes with published pathogenic variants resulting in ocular manifestations in humans. 1P5CDH, Δ^1^-pyrroline-5-carboxylate dehydrogenase; AGT, alanine:glyoxylate aminotransferase; AspAT, aspartate aminotransferase; CAT, catalase; DAAO, D-amino acid oxidase; DASPO, D-aspartate oxidase; EPHX2, epoxide hydrolase 2; GO, glycolate oxidase; GRHPR, glyoxylate and hydroxypyruvate reductase; GSH, glutathione; GSTK, glutathione transferase kappa; HOGA, 4-hydroxy-2-oxoglutarate aldolase; HPOX, hydroxyproline oxidase; LDH, lactate dehydrogenase; MGST, microsomal glutathione S-transferase; PRDX5, peroxiredoxin 5; SOD1, superoxide dismutase. The figure was partly generated using Servier Medical Art.

Glyoxylate is a highly reactive two carbon aldehyde form primarily from metabolisms of plant-source glycolate by peroxisomal glycolate oxidase (GO) and animal-source hydroxyproline by sequential breakdown via hydroxyproline oxidase (HPOX), Δ^1^-pyrroline-5-carboxylate dehydrogenase (1P5CDH), aspartate aminotransferase (AspAT), and 4-hydroxy-2-oxoglutarate aldolase (HOGA) (Li et al., 2016; Wang et al., 2020). Glyoxylate is readily metabolized by dehydrogenase and oxidases to oxalate which then crystalizes with calcium to form calcium oxalate stone, a common source of kidney stones (Tsujihata, 2008). Since glyoxylate accumulation is correlated with dietary sources and intake, multiple detoxification mechanisms involving peroxisome and/or mitochondria are evolved depending on the food sources of the organisms (Danpure, 1997; Birdsey et al., 2005; Martin, 2010). Three enzymes are involved in the detoxification of glyoxylate: 1) peroxisomal and mitochondrial alanine:glyoxylate aminotransferase (AGT, encoded by the gene *AGXT*), 2) mitochondrial and cytosolic glyoxylate reductase/hydroxypyruvate reductase (GRHPR), and 3) cytosolic lactate dehydrogenase (LDH) (Figure 5). In contrast to rat where AGT is localized to both peroxisome and mitochondria, human AGT is exclusively expressed in peroxisome (Danpure, 1997)**.** AGT catalyzes the transamination of glyoxylate and L-alanine to form pyruvate and glycine (Cellini et al., 2007)**.** Cellular expressions of AGT in retina and RPE has not been elucidated.

Peroxisomes is also engaged in the catabolism of D-amino acids (Figure 5). Two stereospecific enzymes are localized to peroxisomes: 1) D-amino acid oxidase (DAAO/DAO) and 2) D-aspartate oxidase (DASPO/DDO) (Van Veldhoven et al., 1991; Pollegioni et al., 2018). DAAO have a wide substrate specificity; however, it prefers bulky hydrophobic D-amino acids (D-DOPA, D-Tyr, D-Phe, D-Trp) while having limited catalytic activity for small uncharged D-amino acids (D-Cys, D-Ala, D-Pro, D-Ser) (Kawazoe et al., 2007; Frattini et al., 2011; Shibuya et al., 2013; Murtas et al., 2017). DAAO does not actively metabolize glycine which explain the negligible contribution of glyoxylate from DAAO-mediated glycine degradation (Thureen et al., 1995; Molla et al., 2006; Murtas et al., 2017)**.** DASPO have high affinity for acidic D-amino acids (D-Asp, NMDA, D-Glu) followed by polar D-amino acids (D-Gln, D-Asn) (Pollegioni et al., 2021). DAAO is expressed in RPE and Müller cells (Beard et al., 1988); whereas DASPO retinal cell expression is unknown.

### 2.4 Anabolic and catabolic function: ROS/RNS metabolism

As its name defined, the core feature of peroxisome is the production of reactive oxygen species (ROS) and reactive nitrogen species (RNS) (De Duve and Baudhuin, 1966), which may cause biomolecular damage and cell death. As an organelle with high productions of ROS/RNS species, naturally, peroxisomes are equipped with an arsenal of antioxidant enzymes and non-enzymatic free radical scavengers (Figure 5). To-date, there is no consensus regarding peroxisome’s overall contribution as a net source of cellular ROS/RNS or sink for ROS/RNS detoxification (Fransen et al., 2013). Cellular reduction-oxidation (redox) state is dependent on several factors including the type, concentration, localization, synthetic rate, and degradation rate of oxidants formed (Trachootham et al., 2008; Forman et al., 2010). The magnitude of oxidative stress can have divergent effects on cellular fates where at high levels can induced irreversible damage to lipids, protein, and DNA resulting in apoptosis, but at low levels oxidants act as signaling messengers to promote cell survival and proliferation (Jones and Go, 2010; Holmstrom and Finkel, 2014).

The most abundant oxidants produced in peroxisomes is hydrogen peroxide (H_2_O_2_) because of flavin-containing oxidases that reduce molecular oxygen (O_2_) to H_2_O_2_ (Antonenkov et al., 2010). Peroxisomes account up to 35% of total hydrogen peroxide generation in the mammalian tissues and 20% of total cellular oxygen consumption (Cipolla and Lodhi, 2017). In human peroxisomes, H_2_O_2_ is degraded by catalase (CAT) and peroxiredoxin 5 (PRDX5). CAT is the most abundant peroxisomal antioxidant enzyme that is highly expressed in RPE as compared to neural retina. CAT activity exhibits a diurnal pattern where post-translational modification of CAT increases antioxidant activity in the morning during peak VLCFA β-oxidation. While PRDX5 can assist in H_2_O_2_ degradation, it is limited due to the small rate constant (Knoops et al., 2011). Superoxide anion radical (O_2_^•–^) and nitric oxide radical (^•^NO) are produced by xanthine oxidase and the inducible form of nitric oxide synthase. While superoxide is degraded by superoxide dismutase 1 (SOD1), ^•^NO is thermodynamically favored to form peroxynitrite (ONOO^−^) with superoxide anion radical after which ONOO^−^is predominantly degraded by PRDX5 (Pacher et al., 2007; Trujillo et al., 2007; Antonenkov et al., 2010). Hydroxyl radical (^•^OH) are produced from H_2_O_2_ through Fenton reactions (Winterbourn, 1995). ^•^OH and ^•^NO may target protein cysteine thiol and unsaturated lipids causing cysteine oxidation and lipid peroxidation (Lismont et al., 2015). PRDX5 also degrades alkyl hydroperoxides (ROOH); whereas LOOH and fatty acid epoxides are detoxified by glutathione transferase kappa (GSTK1)/microsomal glutathione S-transferase 1 (MGST1) and epoxide hydrolase 2 (EPHX2) (Summerer et al., 2002; Sala et al., 2003; Knoops et al., 2011). Free radicals may also be non-enzymatically scavenged by glutathione (GSH), ascorbic acid (vitamin C), and plasmalogens (Circu and Aw, 2010; Ivashchenko et al., 2011). While the mechanisms by which GSH and ascorbic acid modulate oxidative stress are debated, plasmalogen quenches lipid peroxidation and protects unsaturated fatty acid from oxidation in the membrane because the vinyl-ether bond of plasmalogen interact faster with singlet oxygen and free radicals than other fatty acids (Broniec et al., 2011; Wallner and Schmitz, 2011).

Redox imbalance is a significant contributor to multiple retinal metabolic diseases (Kowluru and Chan, 2007; Bellezza, 2018; Trachsel-Moncho et al., 2018; Graziosi et al., 2020). Restoring the redox homeostasis preserves retinal neuronal and vascular stability. However, the role of peroxisomal redox contribution to retinopathy is understudied. A recent report has shown that overactivation of peroxisomal oxidation with a gain function of ACOX1 (*ACOX1* ^*N237S*^) causes elevated ROS and retinal glial loss (Chung et al., 2020). Further exploration of peroxisomal redox balance in retinal function is needed.

### 2.5 Anabolic function: Plasmalogen synthesis

Plasmalogen (Pls) are a subclass of glycerophospholipids defined by a vinyl-ether bond at *sn*-1 position of glycerol backbone. Dependent on the polar head group at *sn*-3 position, compositions of fatty acids at the *sn*-1 ether-linkage and *sn*-2 ester-linkage differs. Plasmalogen ethanolamine (PlsEtn) is the predominant subclass in photoreceptor rod inner segment and RPE (Acar et al., 2007; Bretillon et al., 2008; Acar et al., 2012). In the retina and RPE, PlsEtn accounts for 20–40 and 30% of ethanolamine glycerophospholipids, respectively (Heymans et al., 1983; Acar et al., 2007; Acar et al., 2012). PlsEtn is composed of ether-linked saturated fatty acid (16:0/18:0) or monounsaturated fatty acid (18:1n-9/18:1n-7) at sn-1 position and ester-linked PUFA (DHA/20:4n-6—arachidonic acid, ARA) at *sn*-2 position (Acar et al., 2012). Plasmalogen choline (PlsCho) is the predominant subclass in white matter and optic nerve where it accounts for 76 and 36% of total glycerophospholipids, respectively (Han et al., 2001; Acar et al., 2012). PlsCho is composed of ether-linked saturated fatty acid (16:0/18:0) or monounsaturated fatty acid (18:1n-9/18:1n-7) at sn-1 position and ester-linked saturated fatty acid (18:0) at *sn*-2 position (Han et al., 2001; Acar et al., 2012). The concentrated Pls level in RPE is likely to protect against elevated oxidative stress as discussed in previous section.

Peroxisome is critical for initiation of ether phospholipid synthesis as the initial two enzymes in a complex, glycerone-phosphate O-acyltransferase (GNPAT) and alkyl-glyceronephosphate synthase (AGPS) are targeted to inner peroxisomal membrane via PTS2 and PTS1 signal peptide, respectively (Hasan et al., 2013; Fujiki et al., 2014) (Figure 3). Dihydroxyacetone-phosphate (DHAP) is esterified to a long chain fatty acyl-CoA at *sn*-1 position by GNPAT, then AGPS catalyzes the exchange of 1-acyl-DHAP and long chain alcohol to produce 1-alkyl-DHAP. This exchange is essential to ensure synthesis of alk-1′-enyl ether bond by plasmanylethanolamine desaturate (encoded by TMEM189 gene) in ER (Gallego-Garcia et al., 2019; Werner et al., 2020). Long chain alcohol is synthesized by two peroxisomal acyl-CoA reductases: fatty acyl-CoA reductase 1 (FAR1) and fatty acyl-CoA reductase 2 (FAR2) (Cheng and Russell, 2004). FAR1 have high affinity for palmitoyl-CoA (16:0-CoA), stearoyl-CoA (18:0-CoA), and oleoyl-CoA (18:1n-9-CoA); thereby explaining unique composition of saturated and monounsaturated fatty acids at *sn*-1 ether linkage (Bishop and Hajra, 1981). In contrast, FAR2 catalyzes synthesis of long chain fatty alcohol by binding fatty acyl-CoA with >20 carbons (Otsuka et al., 2022). Finally, 1-alkyl-DHAP is reduced by peroxisomal acyl/alkyl DHAP reductase (ADHAPR—encoded by *Dhrs7b*) (Honsho et al., 2020). GNPAT is ubiquitously expressed in neural retina and RPE (Acar et al., 2007; Das et al., 2019). Retinal distributions of AGPS, FAR1, FAR2, and ADHAPR have not yet been characterized.

### 2.6 Anabolic function: Docosahexaenoic acid (DHA) synthesis

DHA is the predominant omega-3 polyunsaturated fatty acids (n-3 PUFA) in retinal phospholipid membrane and its accretion may modulate membrane fluidity which affects assemblies of protein complexes and their retinal activity (Fliesler and Anderson, 1983; Treen et al., 1992; Litman and Mitchell, 1996). In human, DHA accounts for 15.3% of total fatty acids in photoreceptor rod cell outer segments as compared to 1–4% in other tissues (Fliesler and Anderson, 1983; Acar et al., 2012). In contrast to photoreceptor rod cells, the compositions of total DHA and diDHA (unique phospholipids with two ester-linked DHA) species in phosphatidylcholine, phosphatidylethanolamine and phosphatidylserine were approximately 2-fold lower in cone-dominant retinas (Agbaga et al., 2018). The unique DHA accretion in the retina is maintained by three mechanisms: 1) dietary uptake of DHA (Young, 1976; Wang and Anderson, 1993b; Pifferi et al., 2012), 2) recycling of phospholipid DHA in rod outer segments via phagocytosis (Young, 1976; Stinson et al., 1991b; Gordon et al., 1992; Gordon and Bazan, 1993), and 3) synthesis of DHA from dietary precursors including α-linolenic acid (ALA, 18:3n-3), eicosapentaenoic acid (EPA, 20:5n-3), and DPAn-3 (22:5n-3) (Anderson et al., 1990; Wang and Anderson, 1993a; Alvarez et al., 1994; Delton-Vandenbroucke et al., 1997). Although biosynthesis of DHA from dietary n-3 PUFA precursor in retina and RPE is limited, impairment in peroxisomal β-oxidation of THA to DHA was observed in patients with X-linked retinitis pigmentosa (Wetzel et al., 1991; Hoffman et al., 2001).

DHA synthesis was first described to be solely localized to the ER where a series of alternating elongation by ELOVL5/2 and desaturation by FADS1/2 to produce DPAn-3. DPAn-3 is subsequently desaturated to DHA by Δ4-desaturase. However, the presence of Δ4-desaturase in retina is still heavily debated (Metherel and Bazinet, 2019). Since the publication of Voss et al., in 1991, the dogma of DHA synthesis shifted from a localized ER pathway to concerted efforts between ER and peroxisome which is later termed the Sprecher Pathway (Voss et al., 1991; Sprecher et al., 1995) (Figure 3). From fibroblasts of patients with Zellweger and Refsum disease, the final steps of DHA synthesis were characterized as such that DPAn-3 is elongated to tetrapentaenoic acid (TPA, 24:5n-3) by ELOVL2 and desaturated to THA by FADS2 in ER, then transported to peroxisome via ABCD2, where peroxisomal β-oxidation retroconvert THA to DHA for export (Moore et al., 1995; Morita and Imanaka, 2012). The other possibility is that peroxisomal dysfunction also affect the DHA recycling from photoreceptor outer segment shedding, which will be further discussed in Session 3.

Peroxisomal dysfunction in patients causes DHA deficiency in the brain and retina (Martinez, 1992). Lack of DHA contribute to the pathophysiology of multiple retinal disorders (Fu et al., 2019). In retinal diseases including diabetic retinopathy, retinopathy of prematurity (ROP), age-related macular degeneration (AMD) and inherited retinal degenerative diseases, DHA deficiency is observed. However, DHA supplementation shows positive impacts in some but not all studies (Fu et al., 2019).

### 2.7 Anabolic function: Bile acid synthesis

Bile acids, cholic acid (CA) and chenodeoxycholic acid (CDCA), play an important role in homeostasis of cholesterol metabolism in the retina. Unconjugated and taurine-conjugated metabolites of CDCA, ursodeoxycholic acid and tauroursodeoxycholic acid, are neuroprotective of photoreceptors and ganglion cells (Daruich et al., 2019; Win et al., 2021). Classic bile acid synthesis commenced with conversion of cholesterol to 7α-hydroxycholesterol by 7α-hydroxylase (CYP7A1) leading to production of DHCA and THCA in mitochondria (Chiang, 2004). Then in the ER, CoA activation of DHCA and THCA is catalyzed by bile acid-CoA ligase (BACL) (Wheeler et al., 1997; Falany et al., 2002). DHCA-CoA and THCA-CoA are then transported by ABCD3 into peroxisome where β-oxidation chain shortening produce chenodeoxycholoyl-CoA and choloyl-CoA, respectively (Ferdinandusse et al., 2015) (Figure 4). In contrast to liver, retina predominantly relies on an alternative pathway for bile acid synthesis where mitochondrial CYP27A1 in rod inner segment and ER CYP46A1 in retinal ganglion cells convert cholesterol to 27-hydroxycholesterol and 24-hydroxycholesterol, respectively (Lee et al., 2006; Ramirez et al., 2008; Ishikawa et al., 2016; Mertens et al., 2017). Hydroxylated cholesterol intermediates of CYP27A1 and CYP46A1 are then converted to DHCA and THCA at which classic pathway of CDCA and CA synthesis resume (Omarova et al., 2012; Saadane et al., 2014). Chenodeoxycholoyl-CoA and choloyl-CoA are then conjugated with taurine or glycine by peroxisomal bile acid-CoA:amino acid N-acyltransferase (BAAT) to produce tauro/glycochenodeoxycholate and tauro/glycocholate (Pellicoro et al., 2007).

## 3 Peroxisomal disorders and ocular symptoms

Based on the results of enzyme activity studies and biochemical markers, the peroxisomal disorders was historically divided into three groups: 1) peroxisomal biogenesis disorders (PBD); 2) multiple enzyme deficiencies; and 3) single enzyme/transporter deficiency disorders. With recent development in the understanding of the biochemical and molecular genetic bases of these conditions, a new system has been proposed to divide the PBD into two subtypes: Zellweger Spectrum Disorder (PBD-ZSD) and the rhizomelic chondrodysplasia punctata (RCDP) spectrum (previously known as the multiple enzyme deficiencies). Other conditions such as Heimler Syndrome (HS), which was previously classified as a distinct syndrome consists of sensorineural hearing loss, amelogenesis inperfecta (AI) and nail anomalies with or without visual defect, is now considered as part of the PBD-ZSD (Ratbi et al., 2015). Single enzyme/transporter deficiency disorders such as X-linked adrenoleukodystrophy (X-ALD) and peroxisomal acyl-CoA oxidase (ACOX) deficiency remains a separate entity.

### 3.1 Gene mutations in peroxisomal biogenesis

PBD-ZSD is a group of conditions with a very broad clinical spectrum ranging from neonatal lethal conditions with severe neurological presentation to adult-onset isolated visual impairment, sensorineural hearing loss, adrenal insufficiency, or liver disease. The incidence of PBD-ZSD was estimated to be around 1 per 50,000 births (Braverman et al., 2016). PBD-ZSD is characterized by biochemical findings of elevated plasma VLCFAs (C24 and C26), elevated plasma C24:C22 and C26:C22 ratio, elevated plasma phytanic acid, pristanic acid, pipecolic acid and bile acids concentrations, reduced erythrocyte plasmalogen level, and elevated urinary excretion of dicarboxylic acid (Steinberg et al., 2006; Braverman et al., 2016) (Figure 4). Patients with PBD-ZSD typically carry biallelic (homozygous or compound heterozygous) pathogenic variants in the PEX family genes, which encode proteins involved in peroxisome biogenesis and proliferation. A total of sixteen PEX genes have been identified in humans, and PBD-ZSD have been associated with PEX1, PEX2, PEX3, PEX5, PEX6, PEX10, PEX11β, PEX12, PEX13, PEX14, PEX16, PEX19, and PEX26 (Ebberink et al., 2009; Waterham and Ebberink, 2012; Krause et al., 2013; Fedick et al., 2014; Kettelhut and Thoms, 2014; Komatsuzaki et al., 2015; Konkolova et al., 2015; Renaud et al., 2016; Waterham et al., 2016; Bjorgo et al., 2017; Rydzanicz et al., 2017; Stowe and Agarwal, 2017; Kumar et al., 2018). Patients with loss-of-function PEX gene defects and abolished peroxin activity, are most severe in their clinical presentation. In contrast, variants with residual peroxin function, such as missense variants, result in a milder cellular and clinical phenotype. However, not all missense variants have residual activity, and the clinical course of a patient cannot be predicted by the genetic variants alone.

Diagnosing patients with mild presentation of PBD-ZSD has been challenging due to the diverse and non-specific nature of the symptoms (Enns et al., 2021). Nonetheless, recent expansion in newborn screening programs have included screening of X-ALD using liquid chromatography tandem mass spectroscopy (LC-MS/MS) to detect VLCFA, C26:0-lysophosphatidyl choline (C26:0-LPC), from dried blood spots (Turk et al., 2020). First introduced in 2013, newborn screening for X-ALD is now carried out in 20 States and the District of Columbia with 2 additional states currently running pilot programs ( *https://adrenoleukodystrophy.info/clinical-diagnosis/ald-newborn-screening* ). Therefore, every newborn from these states will have C26:0-LPC testing at birth, which will identify all newborns with PBD-ZSD (Klemp et al., 2021). Concerns have been raised around the “incidental” identification of PBD-ZSD patients through the X-ALD screening due to lack of targeted treatment that modifies the course of PBD-ZSD. Therefore, development of targeted PBD-ZSD treatment is in dire need.

The central nerves system (CNS) manifestations of PBD-ZSD involves structural brain malformations, predominantly involve the neuronal migration defects such as polymicrogyria, heterotopia, agenesis/hypoplasia of the corpus callosum, pachygyria, and hypoplastic olfactory bulb. Cerebral ventricular abnormalities such as colpocephaly and progressive dilatation of the ventricles were also reported (Nakai et al., 1995). Demyelination and global cerebral atrophy have also been reported in both early and late onset cases (Renaud et al., 2016). Ocular phenotypes related to PBD-ZSD includes anterior changes of cornea clouding, cataracts, and Brushfield spots (small, white or greyish/brown spots on the periphery of the iris). Posterior ocular findings include Leber congenital amaurosis (LCA), pigmentary retinopathy, and optic nerve dysplasia (Lambert et al., 1989; Mechaussier et al., 2019). LCA is a severe form of early onset retinal dystrophy. Patients with LCA typically have visual impairment since infancy. Symptoms of LCA include photophobia, nystagmus, keratoconus, and behavior of eye poking and picking (oculo-digital signs). Retinal changes associated with PBD-ZSD involve all retinal layers from RPE to retinal ganglion cells (Haddad et al., 1976), with most severe loss in photoreceptors (Haddad et al., 1976; Kretzer et al., 1981; Garner et al., 1982). These changes are scattered from central to peripheral retina. The optic nerve of patients with PBD-ZSD can be profoundly demyelinated (Thomas et al., 1975). Fundoscopic changes in patients with PBD-ZSD can include retinal arteriolar attenuation, pale optic disc and loss of pigment epithelium most prevalent in the macular area (Folz and Trobe, 1991).

Heimler Syndrome is a condition found to be caused by pathogenic variants in PEX1 and PEX6 genes (Ratbi et al., 2015). It represents the mild end of PBD-ZSD. Long term follow-ups of index cases revealed “salt-and-pepper”-like mottling of RPE and abnormal electroretinogram (ERG) recording in young adulthood after complaints of decreased vision in a female patient. The patient also had cystoid macular edema in keeping with the diagnosis of macular dystrophy (Lima et al., 2011). After evaluating other patients with Heimler syndrome, three additional patients with PEX1 variants and one patient with PEX6 variants were also found to have retinal pigmentary changes (Heimler et al., 1991; Zaki et al., 2016). Given the rarity of these conditions, most literature is constituted with case studies describing the retinal phenotype of PBD-ZSD. Studies focusing on the natural history of retinal dystrophy in PBD-ZSD are required to understand its progressive impact on vision.

### 3.2 Gene mutations in peroxisomal β-oxidation

Peroxisomal acyl-CoA oxidase deficiency disorder caused by biallelic pathogenic variants in *ACOX1* gene and D-bifunctional protein (DBP) deficiency disorder caused by either or both MFE 2-enoyl-CoA hydratase and 3-hydroxyacyl-CoA dehydrogenase deficiency, are disorders of peroxisomal fatty acid β oxidation (Figure 3). They can present with neonatal onset severe neurological sequela with progressive white-matter demyelination, seizures, and cortical malformations. Both conditions were associated with visual impairment, abolished electroretinogram possibly related to retinal degeneration or subretinal pigmentary retinopathy (Suzuki et al., 2002; Kurian et al., 2004; Bae et al., 2020). ACOX1 is abundantly expressed in the RPE as compared to neural retina, whereas the distribution of ACOX2 and ACOX3 in RPE and retinal layers remains unclear (Das et al., 2019). ACOX1 deficiency results in premature death in adolescence and are associated with abnormal retinal pigmentation, retinal degeneration, and optic atrophy (Das et al., 2021). Patients with ACOX1 deficiency have elevated plasma and fibroblast saturated VLCFA and comparable saturated LCFA, BCFA (phytanic acid), and DHCA/THCA to control (Ferdinandusse et al., 2007). MFE-2 is equally or more expressed in the neural retina than RPE, whereas expressions of MFE-1 has yet to be elucidated in the eyes (Das et al., 2019). Severe MFE-2 deficiency may manifest symptoms comparable to that of PBD-ZSD; however milder juvenile onset deficiency may present with abnormal retinal pigmentation with normal visual acuity (Das et al., 2021). Patients with MFE-2 deficiency have elevated levels of saturated VLCFA, BCFA (pristanic acid), and DHCA/THCA and reduced levels of plasma and retinal DHA (Martinez, 1992; Ferdinandusse et al., 2006a). Patients with peroxisomal ACOX deficiency and MFE-2 deficiency show no accumulation of pipecolic acid, with no reduction in erythrocyte plasmalogens. MFE-2 deficiency is associated with increased plasma levels of bile acid intermediates, but ACOX deficiency is not (Ferdinandusse et al., 2007).

SCPx deficiency was first reported in 2006 in a patient with normal level of VLCFA but elevated level of pristanic acid (Ferdinandusse et al., 2006b). The only ocular finding reported was pathological saccadic eye movements indicating potential pathology in the brain stem. Another patient with SCPx deficiency had mild symptoms of nyctalopia. This individual had normal visual acuity. Detailed ocular investigation revealed peripapillary atrophy, irregular autofluorescence associated with the major vascular arcades, with no peripheral pigmentary retinal changes (Horvath et al., 2015; Morarji et al., 2017). ACAA1 deficiency has yet to be associated with human disease.

### 3.3 Gene mutations in peroxisomal α-oxidation

FALDH deficiency leads to Sjögren-Larsson Syndrome, a condition associated with accumulation of phytol. Sjögren-Larsson Syndrome is associated with reduced visual acuity, degeneration of Müller cells in the inner retina, retinal thinning, RPE atrophy, and photoreceptor dysfunction (van den Brink et al., 2004; Nanda and Kovach, 2019). PHYH deficiency is one of the primary causes of Refsum disease (Jansen et al., 1997) (Figure 4). Patient with Refsum disease have elevated levels of phytanic acid in blood and tissues. Clinical symptoms of Refsum disease includes retinitis pigmentosa, miosis with attenuated pupillary light response, iris atrophy, and cataract (Ruether et al., 2010). Phytanic acid exposure cause morphological changes in both fetal bovine and human RPE *in vitro*, mimicking the RPE pathology observed in patients with Refsum’s disease (Bernstein et al., 1992). HACL1 deficiency has yet to be reported in humans; however, animal model suggests that dietary intake of phytol in *Hacl1* ^−/+^ mice exhibited elevated levels of phytanic acid in blood and tissues similar to the biochemical profile of patients with Refsum disease (Mezzar et al., 2017). Interestingly, the human HACL1 is only three amino acids different from its mouse analog. Clinical phenotypes of AMACR deficiency include retinitis pigmentosa and decreased visual acuity (Thompson et al., 2009; Stewart et al., 2011).

### 3.4 Gene mutations in glyoxylate detoxification

Defects in *AGXT* gene results in primary hyperoxaluria type 1, which is an inborn error of metabolism characterized by accumulation of calcium oxalate (Figure 5). This process leads to calcinosis cutis metastatica of the skin, tooth mobility, vascular spasm/occlusion, and nephrocalcinosis. Retinal complications include optic atrophy, retinopathy and choroidal neovascularization (Leumann and Hoppe, 2001; Cochat et al., 2006; Lorenzo et al., 2006). Patients with primary hyperoxaluria type 1 also exhibit crystalline deposit in RPE leading to bilateral vision loss (Roth et al., 2012; Kourti et al., 2020).

### 3.5 Gene mutations in plasmalogen synthesis

GNPAT deficiency results in RCDP type 2 (Wanders et al., 1992) (Figure 3). Patients with GNPAT deficiency have low plasmalogen levels in erythrocytes. They typically have a shortened life expectancy not exceeding a decade (Itzkovitz et al., 2012). Congenital cataracts have been reported (Wanders et al., 1992). AGPS deficiency results in RCDP type 3 and patients exhibited lower levels of plasmalogen in erythrocytes (Wanders et al., 1994). FAR1 deficiency results in autosomal recessive RCDP type 4 (Buchert et al., 2014). FAR1-deficient patients have markedly lower levels of plasmalogens, and congenital cataracts is a cardinal symptom (Buchert et al., 2014). Interestingly, patients with autosomal dominant FAR1 *de novo* variants also shares similar clinical phenotypes including congenital and juvenile cataracts, but a divergent biochemical phenotype of elevated plasmalogens in fibroblasts (Ferdinandusse et al., 2021). FAR1 *de novo* variant disrupted negative feedback regulation leading to un-inhibited biosynthesis of plasmalogen (Ferdinandusse et al., 2021). Deficiency in FAR2 has yet to be associated with human diseases.

### 3.6 Gene mutations in peroxisomal membrane transporter

Although, ABCD1 was shown to be expressed ubiquitously between RPE and neural retina in rodent model (Das et al., 2019), patients with X-ALD do not exhibit direct retinal pathology. The visual impairment in X-ALD patients are caused by two mechanisms: 1) the loss of color vision in a subgroup of X-ALD patients are caused by continuous gene deletions in X chromosome where *ABCD1* gene and red pigment gene are in close proximity (Sack et al., 1989) or by chromosome rearrangements (Alpern et al., 1993), 2) the cortical blindness associated with X-ALD is caused by visual track demyelination, which can subsequently lead to retrograde degeneration of ganglion cells (Kaplan et al., 1995; Ohkuma et al., 2014).

Patients with ABCD3 deficiency have elevated levels of DHCA and THCA, but ocular abnormalities have not yet been identified (Ferdinandusse et al., 2015). Patients with BAAT deficiency exhibited elevated levels of unconjugated bile acids (Carlton et al., 2003). BAAT expression in retinal cells are unknown and patients with BAAT deficiency has no reported ocular abnormalities.

Recently, patients with biallelic pathogenic variants in acyl-CoA binding domain containing protein 5 (ACBD5) gene have been reported (Abu-Safieh et al., 2013; Ferdinandusse et al., 2017; Bartlett et al., 2021). These patients demonstrated progressive neurological deterioration, nystagmus, optic atrophy, cone-rod dystrophy, early onset retinal dystrophy, attenuation of eye vessels, and diffuse granularity of RPE.

### 3.7 Current therapy

Currently, there is no curative or targeted disease modifying treatment for PBD-ZSD. Cholic acid (CA) therapy was approved by Food and Drug Administration (FDA) in March 2015 to treat liver disease in patients with PBD-ZSD. CA has been shown to decrease toxic bile acid intermediates, improve transaminase levels, and reduce liver inflammation, with improvement in growth parameters in pediatric populations (Anderson et al., 2021). Since PBD-ZSD patients have markedly low level of DHA (Janssen et al., 2000), trials of DHA supplementation were made as an attempt to restore the neurological, ocular and hepatic function in PBD-ZSD patients. M. Martinez conducted a cohort study in the 1990s treating 20 PBD-ZSD patients with DHA supplementation. Patients were treated with variable doses of DHA ethyl ether (depends on age and blood DHA level) between 9 months and 9 years and more than half of the patients not only had improved biochemical markers such as reduced VLCFA and increased erythrocyte plasmalogen level, but also improved brain and retinal function compared to pre-treatment (Martinez, 2001). Nonetheless, the clinical improvement was not subjectively quantified. A follow up study focusing on the visual function of this cohort demonstrated resolution of nystagmus, improvement of ERG recording, and amelioration of visual behaviors in some cases and stabilization in the rest. The improvement was better appreciated in patients who had baseline ophthalmological assessment (Noguer and Martinez, 2010). Based on this preliminary work, a randomized double-blind case control trial was conducted supplementing a fixed dose (100 mg/kg/d) of DHA triglyceride (47% DHA) and arachidonic acid (ARA; 20:4n-6) triglyceride (46% ARA) (Paker et al., 2010). This is a short-term study in 50 patients (25 in treatment group and 25 in control) with follow ups in 1 year. The study also focused exclusively on quantitative biometrics of patients such as ERG changes, weight, height and biochemical markers such as VLCFA, plasmalogen and liver enzymes. Given the short-term intervention and the broad spectrum of clinical variability of PBD-ZSD patients, it was not surprising that no statistically significant changes were obtained between the two groups. At this time, there is very limited evidence supporting the efficacy of clinical use of DHA in patients with PBD-ZSD.

## 4 Peroxisomes and other cell organelles

Peroxisomes are highly dependent on the interaction with other organelles (ER, mitochondria and lysosomes) in order to perform proper metabolism. For example, some of the peroxisomal substrates (e.g., VLCFAs) are synthesized and elongated from shorter chain fatty acids (SCFAs) in ER. In addition, the end products of peroxisomal β-oxidation subsequently enter mitochondria for metabolism and energy production.

### 4.1 Peroxisomes and ER

**FIGURE 6:**
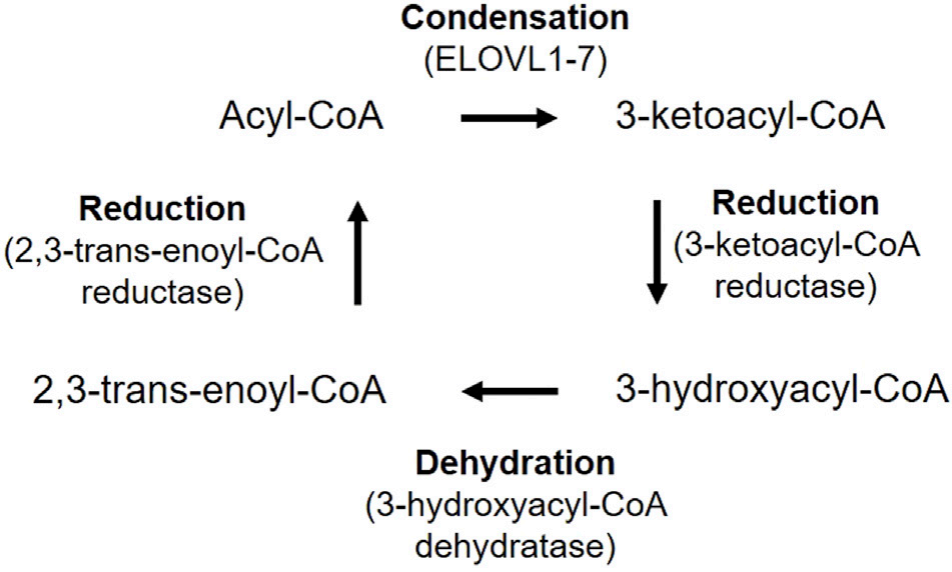
VLCFA biosynthesis in mammalians. Acyl-CoA is first condensed with malonyl-CoA to produce 3-ketoacyl-CoA, catalyzed by fatty acid elongase (ELOVL1-7). 3-ketoacyl-CoA is reduced to 3-hydroxyacyl-CoA by 3-ketoacyl-CoA reductase. 3-Hydroxyacyl-CoA is then dehydrated by 3-hydroxyacyl-CoA dehydratase and generate 2,3-trans-enoyl-CoA. Finally, 2,3-hydroxyacyl-CoA is reduced to an acyl-CoA by 2,3-trans-enoyl-CoA reductase, with two more carbon chain units than the original acyl-CoA.

Most of VLCFAs are not derived from dietary source, instead, FAs are elongated in ER by cycling through a four-step process (condensation, reduction, dehydration and reduction) (Figure 6) (Kihara, 2012). Acyl-CoA is elongated to have two more carbon units in each cycle. The FA elongases ELOVL1-7 have characteristic substrate specificity (Kihara, 2012). ELOVL1,3,4,6 and 7 elongate SFAs and MUFAs, while ELOLV2 and 5 are strictly PUFA-specific. VLCPUFAs uniquely exist in mammalian retina, brain, and sperm (Kihara, 2012). ELOLV4 is critical for the synthesis of VLCFAs and expressed highest in the retina (Agbaga et al., 2010; Kihara, 2012). Mutations in the *ELOVL4* gene leads to Stargardt disease type 3 that causes vision loss starting around 14 years old (Edwards et al., 1999; Agbaga et al., 2010). Loss of photoreceptor ELOLV4 in mice causes significant decrease in retinal glycero-phospholipids containing VLCPUFAs, abnormal accumulation of lipid droplets and lipofuscin-like granules, as well as decreased retinal neuronal responses by ERG (Harkewicz et al., 2012). In addition, adiponectin receptor 1 (ADIPOR1), which is predominantly expressed in photoreceptors (Fu et al., 2017), may recycle VLCPUFAs (containing DHA C22:6) from shed photoreceptor apical disk membrane back to photoreceptor inner segments and further elongate in ER, thus preserves photoreceptor cell survival (Rice et al., 2015). The VAMP-associated proteins (VAP)-ACBD5 complex acts as the primary ER-peroxisome tether, and loss of VAP or ACBD5 increases peroxisome mobility. VAP-ACBD5-mediated contact between the ER and peroxisomes is also important for peroxisome growth and lipid homeostasis (Costello et al., 2017; Hua et al., 2017).

In addition, plasmalogen synthesis starts in peroxisomes and completes in ER (Nagan and Zoeller, 2001; Braverman and Moser, 2012). The initial two steps are catalyzed by peroxisomal matrix enzymes dihydroxyacetone phosphate acyltransferase (DHAPAT/GNPAT) for transfer of acyl-DHAP across the enzyme active sites, and AGPS for the exchange of the acyl group (fatty acid) for an alkyl group (fatty alcohol, supplied by FAR1). Alkyl-DHAP is further reduced and plasmalogen synthesis proceeds in the ER (Figure 2).

Moreover, ER is involved in peroxisome biogenesis. Peroxisomal membrane proteins (PMP) are thought to be first inserted in ER and subsequently exit from ER to peroxisomes (Dimitrov et al., 2013). It has also been proposed that peroxisomes originate from ER and the avoidance of toxic lipid by-products is the driving force for the separation of the peroxisomes (Gabaldon, 2014). In mammalian cells, PEX16 with an appended NH_2_-terminal type I signal anchor sequence moves to peroxisomes after being cotranslationally synthesized in the ER (Kim et al., 2006). In mouse dendritic cells, PEX13 and PMP70 are present in the ER domain with a peroxisomal reticulum from which mature peroxisomes are derived (Geuze et al., 2003). Meanwhile, the peroxisome biogenesis factors PEX19 and PEX3 cooperate and mediates the posttranslational targeting of proteins such as UBXD8, a lipid droplet-destined membrane protein and reticulon homology domain-containing protein to ER (Schrul and Kopito, 2016; Yamamoto and Sakisaka, 2018). Therefore, the close connection between peroxisome and ER is evident.

### 4.2 Peroxisomes and lysosomes

Peroxisomal substrates such as VLCFAs can also be generated from lysosomal degradation of different lipid species. Rod photoreceptor outer segment is rich in VLCPUFA and LCPUFA (DHA and ARA), which comprise ∼60% of total phospholipids (Stinson et al., 1991a). To maintain normal retinal function, there is a continuous shedding of photoreceptor outer segment on daily basis (Fliesler & Anderson, 1983). The sheded outer segments are ingested by RPE via phagocytosis and packaged into membrane-bound phagosomes, which are then fused with lysosomes for subsequent degradation (Bosch et al., 1993; Ferrington et al., 2016). It has been reported that there is a dynamic membrane contact between peroxisomes and lysosomes mediated by lysosomal Synaptotagmin VII (Syt7) binding to the lipid PI(4,5)P_2_ on peroxisomal membrane, and the organelle contact facilitates the transport of cholesterol from lysosomes to peroxisomes (Chu et al., 2015). Cholesterol is accumulated in cells and animal models of peroxisomal disorders (Chu et al., 2015). These findings suggest the potential involvement of peroxisomes in VLCFAs recycling during photoreceptor outer segment shedding. Although RPE immediately recycles DHA released from the disc membrane during degradation back to photoreceptors for disc membrane synthesis (Bazan et al., 1992), the involvement of peroxisome in this process is yet to be characterized. Moreover, compromised lysosomes with impaired degradative enzyme activity leads to lipofuscin-like autofluorescence in RPE (Guha et al., 2014). Understanding of the interactions between peroxisome and lysosome will uncover the basic lipid biology and balance in retina.

### 4.3 Peroxisomes and mitochondria

Peroxisomes and mitochondria tightly interact with each other in fatty acid metabolism and undergo close communication through three major pathways: 1) there is a physical association between these two organelles, and the degree of contact changes dynamically with the cell types and microenvironment (Fransen et al., 2017). For example, the peroxisomal membrane protein PEX34 (conserved to humans) and the outer mitochondrial membrane protein FZO1 (mammalian homologue Mitofusin 2) tether the peroxisome and mitochondria in the yeast (Shai et al., 2018). 2) mitochondria-derived vesicles containing mitochondria-anchored protein ligase that can fuse with a subset of peroxisomes (Neuspiel et al., 2008); 3) peroxisomes and mitochondria communicates through releasing of biological messengers such as ROS, lipids and NAD^+^ etc.

VLCFAs, which cannot be degraded in mitochondria, need to be shortened by peroxisome β-oxidation. Shortened VLCFAs are further oxidized in mitochondria for energy production. Peroxisomal β-oxidation can also compensate defective mitochondrial fatty oxidation by breaking down medium-chain and long-chain fatty acids (Violante et al., 2019; Ranea-Robles et al., 2021). Studies of RPE from AMD vs control patients indicated that proliferation of peroxisomes is accompanied with mitochondrial abnormalities (Feher et al., 2006). Abnormal peroxisomal metabolism and redox status can rapidly disturb mitochondrial redox state. The antioxidant enzyme CAT is mainly localized in peroxisomes. Deficiency of CAT increases redox state of mitochondria and peroxisome-derived oxidative stress causes mitochondrial redox imbalance (Ivashchenko et al., 2011). Retinal transfection of AAV-expressing CAT and superoxide dismutases 2 (SOD2) prolonged cone photoreceptor survival in mice during retinal degeneration (Xiong et al., 2015), and AAV-CAT also exerts neuroprotective effects on retinal ganglion cells in ischemia-induced retinal injury in rats (Chen and Tang, 2011).

In addition, peroxisomal oxidation also regulates lipid composition in mitochondria membrane, which in turn regulates mitochondrial dynamics and ATP production (Petrushka et al., 1959; Hatch, 2004). Oxidative stress induces release of cytochrome *c* from mitochondria, and cytochrome *c* cleaves plasmenylcholine (plasmalogens of the phosphocholine head group class) in membrane bilayers (Jenkins et al., 2018). Loss of plasmalogens in mitochondrial membrane causes inefficient respiration (Kimura et al., 2019). As VLCPUFAs are recycled back to the retina during photoreceptor outer segment renewal (Bazan et al., 1992; Gordon et al., 1992), mitochondrial DHA and ARA content could also be potentially modulated by peroxisomal oxidation. Both dietary DHA (no ARA) and ARA (no DHA) in rats profoundly alters cardiac mitochondrial phospholipid fatty acid compositions, and suppresses Ca^2+^-induced opening of the mitochondrial permeability transition pore (MPTP), which causes cell death (Khairallah et al., 2010; Khairallah et al., 2012); dietary DHA (no ARA) also depletes cardiac mitochondrial ARA content (Khairallah et al., 2012). These findings suggest that peroxisomal oxidation may control mitochondrial membrane composition and activity via VLCPUFA recycling. More recently, it has been reported that adipose specific knockout of the peroxisomal biogenesis factor PEX16 or the plasmalogen synthetic enzyme GNPAT blocks cold-induced mitochondrial fission, decreases mitochondrial copy number and causes abnormal mitochondrial function. Dietary plasmalogen increases mitochondrial copy number, improves mitochondria function in mice with *Pex16* deficiency (Park et al., 2019). This work provides direct evidence of peroxisome-derived ether lipid regulating mitochondrial activity.

Moreover, mitochondrial respiration is also critical to reoxidize NADH formed in peroxisomes and maintain peroxisomal β-oxidation (Wanders et al., 2015). Altered NAD (+)/NADH redox state with age-dependent increases in intracellular NADH and decreases in NAD (+) content is shown in human brain (Zhu et al., 2015), reflecting declined mitochondrial function. NAD (+) levels also decrease in mouse RPE cells with aging (Jadeja et al., 2018). Mutations of gene encoding nicotinamide mononucleotide adenylyltransferase 1 involved in NAD (+) biosynthesis reduce the enzymatic activity and causes blindness within the first year after birth (Koenekoop et al., 2012). Taken together, peroxisomal metabolic and redox homeostasis is important to maintain mitochondrial function. Further understanding of peroxisomal and mitochondrial interaction will provide more clues to understand cellular activity in healthy and diseased retinas.

## 5 Knowledge from the brain

Although there are limited studies regarding the disease pathogenesis of peroxisome-related retinal dysfunction, we could gain some knowledge from the brain investigations as retina is part of the CNS (London et al., 2013). Peroxisomal dysfunction leads to compromised antioxidant defense with decreased CAT expression, attenuated D-serine degradation, disrupted lipid composition in myelin sheath and cellular membranes in the CNS (Uzor et al., 2020), which in turn cause abnormalities in the myelinated axons and synaptic transmission (Berger et al., 2016). Accumulated VLCFAs and decreased plasmalogen ethanolamine levels are observed in the brain of patients with Alzheimer’s disease (Ginsberg et al., 1995; Kou et al., 2011). Reduced DHA, ARA and plasmalogen ethanolamine levels are found in the brain of patients with Parkinson’s disease, and supplementation of DHA-containing plasmalogen precursor protects against striatal dopamine loss in mice modeling Parkinson’s disease (Fabelo et al., 2011; Miville-Godbout et al., 2017). These findings suggest an essential role of peroxisomes and the related lipid metabolism in maintaining brain health.

The contribution of peroxisomes to brain function could be cell-dependent. In mice, dysfunction of peroxisome in neural cells leads to abnormal cortical neuronal migration and maturation of the cerebellum (Janssen et al., 2003; Krysko et al., 2007; Muller et al., 2011). Peroxisome deficiency in astrocytes disturbs brain-derived neurotrophic factor levels and dysregulates axogenesis of the hippocampus neurons (Abe et al., 2020), suggesting that astrocytic peroxisomes modulate neuronal integrity. Moreover, microglial peroxisomal dysfunction induces an inflammatory activated and proliferative state in the mouse brain, but does not affect neuronal health up to one-year old and the long-term impacts is yet to be evaluated (Beckers et al., 2019). Therefore, it would be important to uncover the contribution of neuronal and glial peroxisomal activity to neuronal function in the CNS.

## 6 Future perspectives

Defective peroxisomal function causes disturbed retinal homeostasis and retinal degeneration. However, the mechanisms behind are largely unknown. We speculate that the disrupted lipid metabolism and redox balance typically regulated through the interaction between peroxisomes, lysosomes, mitochondria and ER may contribute to the disease development and progression. Further investigations in the pathophysiology and natural history of peroxisomeal disorder associated retinal dystrophy will greatly advance the potential for therapeutic development.

### 6.1 Retinal lipid homeostasis and alterations

The retinal lipid homeostasis is essential in maintaining normal retinal neuronal and vascular function (Fu et al., 2019; Fliesler, 2021). As there are extensive lipid alterations accompanied with peroxisomal dysfunction, it is important to know that whether all or certain altered lipids are the significant contributors to the development of retinal diseases. Lipids are important membrane components of cells and organelles. Change in lipid composition alters organelle function and cell status. In addition, lipids and their downstream lipid metabolites can function as signaling molecules to induce various cellular responses in metabolism, inflammation, cell survival and apoptosis. Moreover, fatty acids such as palmitate are also possible fuel substrates for photoreceptor, but other type and source of fatty acids are understudied (Joyal et al., 2016). Supplementation of specific deficient lipids and modulating the downstream targeting pathways may be protective against retinal degeneration.

Furthermore, the interaction between RPE, Müller glia, and neuronal cells in retina is also important for the modulation of lipid availability and clearance of neighboring photoreceptors (Fu et al., 2021). For example, disrupted lipid homeostasis in RPE causes lipid droplet formation and RPE dysfunction with aging (Yako et al., 2022). Interestingly, peroxisomes and lipid droplets have physical interaction within cells. Peroxisomal β-oxidation-derived ROS regulate the levels of adipose triglyceride lipase, which control lipolysis of intracellular lipids (Ding et al., 2021). Therefore, increasing peroxisomal activity may decrease lipid droplets in RPE, preserve RPE function and in turn photoreceptor health. Fatty acid profiling focuses on the entire tissue and radiolabeled lipid tracing aids the understanding of the kinetic incorporation, turnover and loss of fatty acids in the retina (Rodriguez de Turco et al., 1990; Gordon and Bazan, 1993; Santos et al., 1995). However, the knowledge of cell-specific uses of lipids and the interactions among different lipid metabolic pathways are limited. The feasibility of single-cell transcriptomics and cell-specific lipidomics in retinal research may provide insight to the cell specific lipid and fatty acid metabolism. Furthermore, progress in characterizing dynamic processing of fatty acids in retina is often limited by the high cost of radiotracer and retinal tissue size. Recently, a cost-effective, tracer-free novel methods using compound-specific isotope analysis (CSIA) has been established to trace fatty acid metabolism by utilizing food sources differing in natural carbon isotope abundance. Briefly, because the carbon isotopic composition of a molecule is conserved following dietary intake, highly precise measurement of carbon 13 (^13^C) abundance can provide insights to the dietary source that contributed to the incorporation of ^13^C to an endogenous molecule. Therefore, by designing well-controlled dietary switch experiments that switches diets made from foods less enriched in ^13^C foods (C3 plants) to foods more enriched in ^13^C (C4 plants), fatty acid incorporation, turnover, and synthesis can be measured as effectively as radiotracer experiments (Lacombe et al., 2020; Lacombe and Bazinet, 2021). Moreover, due to the high precision of ^13^C measurements by gas chromatography-isotope ratio mass spectrometry (GC-IRMS), cell-specific retinal extract can be quantified.

### 6.2 Implications of peroxisomes on other retinal disorders

Altered peroxisomal function has been implicated in age-related neurodegenerative diseases and diabetes-related complications (Cipolla and Lodhi, 2017). In RPE from AMD vs control patients, mitochondrial changes are associated with proliferation of peroxisomes (Feher et al., 2006). However, it is unclear that whether this mitochondrial response is protective or detrimental. Further investigation is required.

Furthermore, the knowledge of DHA metabolism and the application of dietary DHA for prevention of retinal dysfunction is translatable to a number of retinal diseases. Some (but not all) clinical trials showed that fish oil (rich in DHA and EPA) supplementation reduces risk for severe ROP (Pawlik et al., 2011; Beken et al., 2014; Pawlik et al., 2014; Molloy et al., 2016; Najm et al., 2017). Fish oil supplementation or dietary intake of food rich in DHA decreased the risk for AMD in a number of trials (Sangiovanni et al., 2009; Tan et al., 2009; Christen et al., 2011; Ho et al., 2011). However, dietary supplementation of DHA (350 mg/day) and EPA (650 mg/day) did not reduce the risk of advanced AMD in Age-Related Eye Disease Studies (AREDS/AREDS2) (Age-Related Eye Disease Study 2 Research Group, 2013). The reasons for the inconsistent observations are unclear. It has been noted that DHA and EPA supplements may lower circulating ARA levels (D’ascenzo et al., 2014; Zhao et al., 2015) and decrease the ARA/DHA ratio in preterm infants (Najm et al., 2017). Low serum ARA level in the postnatal period is strongly associated with development of clinically significant ROP (Lofqvist et al., 2018) and sufficient ARA is required for DHA to protect against severe ROP (Hellstrom et al., 2021). In addition, DHA- and ARA-oxylipins generated via cytochrome P450 oxidases exaggerates while DHA- and ARA-derived oxylipins via lipoxygenases inhibit pathological retinal neovascularization (Sapieha et al., 2011; Gong et al., 2016; Gong et al., 2017). Therefore, metabolic imbalance among these pathways may also contribute to the inconsistent observations among different trials.

In conclusion, understanding of retinal cell-specific peroxisomal function, VLCFA metabolism and natural history of retinal dysfunction in peroxisomal disorders is a key to development of disease modifying therapeutics. Given the involvement of peroxisomal dysfunction in various retinal pathophysiology, this therapeutic intervention is likely to benefit common multifactorial retinal degenerative disorders as well.
